# Self-reported halitosis and oral health related quality of life in adolescent students from a suburban community in Nigeria

**DOI:** 10.4314/ahs.v20i4.62

**Published:** 2020-12

**Authors:** Omolola Alade, Ebenezer Ajoloko, Aderonke Dedeke, Omolara Uti, Oyinkansola Sofola

**Affiliations:** 1 Obafemi Awolowo University, Department of Preventive and Community Dentistry; 2 Lagos University Teaching Hospital, Department of Oral & Maxillofacial Surgery; 3 University of Lagos College of Medicine, Department of Preventive Dentistry

**Keywords:** Halitosis, oral health, quality of life, adolescent

## Abstract

**Background:**

Halitosis is an important cause of impaired quality of life in adolescents. Little is known about the prevalence of self-reported halitosis in adolescents in Nigeria and the extent to which self-reported halitosis impairs their oral health related quality of life.

**Objectives:**

To determine the prevalence and impact of self-reported halitosis on the oral health related quality of life of adolescent students in a suburban community in Nigeria.

**Methods:**

An analytical cross-sectional study. Pre-tested self-administered pro-forma was used to obtain the adolescents' demographic data and their self-perception of halitosis. The Oral Health Impact Profile (OHIP-14) was used to assess the adolescents' OHRQoL. The Mann-Whitney U test was used to compare the median OHIP-14 scores between adolescents who reported halitosis and those who did not. The level of significance was set at p < 0.05. Ethics approval for this study was obtained from the Health Research and Ethics Committee of the Lagos University Teaching Hospital.

**Results:**

A total of 361 adolescents aged 10 – 19 years (mean age 14.1 ± 1.79 years) took part in the study. Of these, 32.7% (n=118) had self-reported halitosis. The median OHIP-14 score among adolescents with self-reported halitosis was 3 (0–9) while those who did not report halitosis had a median OHIP-14 score of 0 (0 – 5). This difference was statistically significant (p < 0.0001).

**Conclusion:**

Self-reported halitosis significantly impaired the oral health related quality of life of the adolescents.

## Introduction

Halitosis can simply be defined as an unpleasant odour that emanates from the mouth. 1, 2 It has been classified into three main categories: genuine, pseudo, and halitophobia. Halitosis is genuine if it is beyond a socially acceptable level; pseudo if it is only perceived by the affected person while halitophobia is halitosis that the affected person perceives as persisting after treatment. 1, 2 Halitosis ranks as the third most common presenting complaint of dental patients following dental caries and periodontal diseases. 2 It may be assessed both objectively and subjectively. However, the subjective assessment may be more relevant in assessing the impact of halitosis on people's quality of lives than an objective assessment. 3 Furthermore, a systematic review showed no significant difference in the prevalence of halitosis as measured by organoleptic method, level of volatile Sulphur compounds and self-report. 4

The reported prevalence of self-reported halitosis in adolescents varies quite widely ranging from 23.6% – 54.7%. 5–8 In Nigeria, a hospital-based study conducted on self-reported halitosis revealed a prevalence of 14.8% and 17.1%. 1, 9

Halitosis has been shown to impair health because it affects overall health, well-being and self-esteem negatively.^10^, ^11^ Studies also show that halitosis impairs social relations with others. 12–14 Halitosis has been described as being the most important condition to create a bad first impression on others. 3 In another study among adolescent males in Brazil, the respondents reported feelings ranging from shame, tenseness to avoidance of close relationships if they felt they suffered from halitosis. 15 Furthermore, previous studies show that adolescents in New Zealand and Brazil rate halitosis as the most important oral condition impacting their oral health related quality of life. 5, 16, 17 This study therefore sought to add to this knowledge base by describing the prevalence of self-reported halitosis among adolescent students and the extent to which it impairs their oral health related quality of life (OHRQoL) in a suburban community in Nigeria.

## Materials and methods

An analytical cross-sectional study was conducted among adolescent students in Pakoto community of Ogun State, Nigeria. The sample size formula for comparison of a continuous variable outcome between 2 groups yielded a total minimum sample size of 353. The parameters used were level of significance of 0.05, power of 80%, mean difference of 1.7 and standard deviation of 5.7. 18 The secondary schools in this community were stratified into private and public schools. Two schools were randomly selected from each stratum to give a total of 4 schools. From each school, about 90 students were recruited into the study evenly spread across the 6 secondary school classes (Junior secondary classes I – III and senior secondary classes I – III). The inclusion criteria were students aged 10 – 19 years who attended secondary schools in Pakoto community. Students with pre-existing systemic conditions, dental caries, orofacial pain, malocclusion, traumatic dental injury and oral mucosal lesions were excluded from the study. Pre-tested self-administered questionnaires were used to obtain respondents' demographic data and their self-perception of halitosis. Respondents were deemed to have reported halitosis if they answered, “Yes”, to the question, “Do you suffer from bad breath?”. The Oral Health Impact Profile -14 (OHIP-14) was used to assess the adolescents' oral health related quality of life (OHRQoL). The OHIP-14 has been validated as a measure of OHRQoL among adolescents in Nigeria^18^. The OHIP-14 scores responses on a Likert scale which ranges from 0 = “Never” to 4 = “Very Often”. Therefore, higher scores on the OHIP-14 equated to poorer OHRQoL.

The background characteristics were presented with mean (SD), frequencies and proportions. The Student's Independent t-test and Chi square test were used to compare the background characteristics of adolescents who reported halitosis and those who did not. The OHRQoL scores were not normally distributed and so were presented with median (IQR) and graphically, with box plots. The Mann-Whitney U test was used to compare the median OHRQoL scores of adolescents who reported halitosis and those who did not. The Mann-Whitney U Test is not sensitive to outliers because all the OHIP-14 scores are ranked. The effect size of the Mann-Whitney U test and its 95% confidence was calculated as described by Conroy (2012).^19^ The level of significance was set at p<0.05. Data analysis was done using STATA 13.1 (Stata Corp. 2013. Statistical Software. Release 13. College Station, TX, USA).

The principals of the schools gave approval for the study. Informed consent was obtained from parents/guardians. Students aged 18 years and above also gave informed consent while assent to take part in the study was obtained from students less than 18 years old. Ethics approval for this study was obtained from the Health Research and Ethics Committee of the Lagos University Teaching Hospital.

## Results

A total of 361 adolescents took part in this study, 146 boys (40.4%) and 215 girls (59.6%). The adolescents had an age range of 10 – 19 years with a mean age of 14.1 ± 1.79 years. Table 1 shows that most of them, 303 (83.9%) attended public schools and 313 (86.7%) of the adolescents reported that both parents were their caregivers. Close to two thirds of the adolescents (60.4%) reported that they brushed their teeth once daily. Only 8.9% and 2.2% of the adolescents gave a positive history of alcohol and tobacco use respectively. Less than 20% (17.2%) of the adolescents reported that they had ever visited a dentist.

**Table 1:** Background characteristics of study participants by self-reported halitosis status (N=361)

Characteristic	Total Study Population (N=361) N (%) or Mean (SD)	Adolescents with Self-Reported Halitosis (N=113) N (%) or Mean (SD)	Adolescents without Self-Reported Halitosis (N=248) N (%) or Mean (SD)	p value
Age (years)	14.1 1.79	13.9 1.65	14.2 1.85	0.09
**Gender**				
Female	215 (59.6)	61 (54.0)	154 (62.1)	0.15
Male	146 (40.4)	52 (46.0)	94 (37.9)	
**Type of** **School**				
Public	303 (83.9)	101 (89.4)	202 (81.5)	0.06
Private	58 (16.1)	12 (10.6)	46 (18.5)	
**Caregiver**				
Both Parents	313 (86.7)	94 (83.2)	219 (88.3)	0.10
Single Parent	34 (9.4)	11 (9.7)	23 (9.3)	
Others	14 (3.9)	8 (7.1)	6 (2.4)	
**Daily** **Brushing** **Frequency**				
Once	218 (60.4)	81 (71.7)	137 (55.2)	0.02
Twice	136 (37.7)	32 (28.3)	104 (41.9)	
>Twice	7 (1.9)	0 (0.0)	7 (2.8)	
**Previous Visit** **to the Dentist**				
Yes	62 (17.2)	22 (19.5)	40 (16.1)	0.44
None	299 (82.8)	91 (80.5)	208 (83.9)	
**Alcohol Use**				
Yes	32 (8.9)	14 (12.4)	18 (7.3)	0.11
No	329 (91.1)	99 (87.6)	230 (92.7)	
**Tobacco Use**				
Yes	8 (2.2)	4 (3.5)	4 (1.6)	0.25
No	353 (97.8)	109 (96.5)	244 (98.4)	

The study further revealed that 32.7% (n=118) of the subjects had self-reported halitosis. There were no statistically significant differences between adolescents who reported halitosis and those who did not concerning age, gender, type of school, caregiver, previous dental visit, alcohol and tobacco use. However, there was a statistically significant difference in the frequency of daily tooth brushing between the 2 groups. (Table 1)

**Figure 1:**
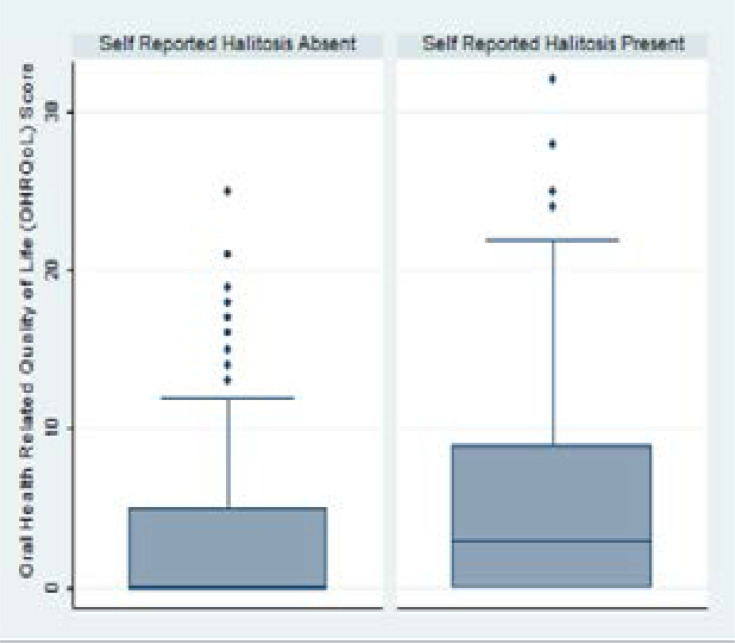
Box Plots of Oral Health Related Quality of Life Score by Halitosis *data more than 1.5 times from the interquartile range are plotted as outliers

The overall OHIP-14 score range was 0 – 32. The overall median OHIP-14 score was 1; interquartile range 0 – 25. Figure 1 shows that the median OHIP-14 score among adolescents with self-reported halitosis was 3 (0–9) while adolescents who did not report halitosis had a median OHIP-14 score was 0 (0 – 5). The Mann-Whitney U test showed that this difference was statistically significant (p < 0.0001), z = -5.31; there was a 66.4% (60.3% – 72.3%) probability that adolescents with self-reported halitosis would have a higher OHIP-14 score than those who did not.

## Discussion

In this study, the prevalence of self-reported halitosis was found to be 32.7%. Studies from various regions of the world show that the prevalence of self-reported halitosis among adolescents varies quite widely. 6–8,16 The lowest prevalence of self-reported halitosis was from a national survey in South Korea, 23.6%. 8 Studies from the southern and southeastern regions of Brazil showed a prevalence of 39.7% and 43.0% respectively.^7^,^16^. While a study of adolescents in a prefecture in Japan recorded the highest prevalence of 54.7%. 6 Thus, the prevalence of 32.7% recorded in this study fell within the range of 23.6 – 54.7% reported from these studies.

The variations in the prevalence of self-reported halitosis can be attributed to the study population; adolescent students may be more inclined to report halitosis than non-students. The prevalence of 23.6% reported from South Korea was from a nationally representative sample of adolescents and may be the closest representation of the true prevalence of self-reported halitosis in adolescents. On the other hand, the prevalence of 39.7% and 43.0% reported from Brazil were from adolescents in schools in Brazil and this may explain the closeness of the results to this study's finding. Furthermore, the phrasing of the question assessing self-reported halitosis (“Subjects were also asked whether they were conscious of self-oral malodour”) in the Japanese study may be responsible for the very high prevalence of 54.7% reported.

This study also found that self-reported halitosis significantly impaired adolescents' oral health related quality of life (p < 0.0001). The median (IQR) OHIP-14 score among adolescents with self-reported halitosis, 3 (0 – 9) was significantly higher than the median score of 0 (0 – 5) observed for adolescents who did not report halitosis. The median OHIP-14 score of 3 among the adolescents who reported halitosis was significantly higher the median of 0 for those who did not.

In previous studies, self-reported halitosis also significantly impaired the oral health related quality of life of adolescents. Studies conducted among adolescents in New Zealand and Brazil, using OHIP-14, also demonstrated that self-reported halitosis significantly impaired oral health related quality of life. 5, 17, Colussi et al.,^5^ found that out of 13 socio-demographic, behavioural and oral factors assessed, only self-reported halitosis and socio-economic status had significant impacts on adolescents' OHRQoL in their study. 5 Similarly, Broughton et al., reported that adolescents with self-reported halitosis had higher impacts on OHRQoL than those without self-reported halitosis. 17 Studies in Chinese and Swedish adults also affirm this finding. 10,20 Thus self-reported halitosis appears to be a condition that cuts across cultures, geographic spaces and significantly impairs adolescents' oral health related quality of life.

The overlap in the interquartile ranges (0 – 9 and 0 – 5) of the adolescents with and without self-reported halitosis respectively suggested that it played a limited role in the OHRQoL of adolescents in this study. However, the 66.4% (60.3% – 72.3%) probability that students with self-reported halitosis would have a higher OHIP-14 score than those who did not corroborated the statistically significant p < 0.0001.

This study's strength lies in the fact that it is the first to report on the impact of halitosis on the oral health related quality of life of adolescents in Nigeria. Adolescence is a peculiarly precarious period when social problems assume epic proportions and given the importance of halitosis on social relationships, it is particularly important to study the impact of halitosis on the OHRQoL in Nigeria with the highest population of adolescents in Africa.

## Limitation

The adolescents studied were adolescents in school. Within the context of Nigeria's educational situation where about 30% of adolescents are out of school, 21 this study fails to capture the full picture of the Nigerian situation. However, this study lays the groundwork for further studies in adolescent oral health which is a neglected area in Nigeria. Furthermore, this was a cross-sectional study and it would be difficult to prove that the impact of OHRQoL observed among these students was solely due to their halitosis status. Thus, we acknowledge that this study has shown association and not causality. However, this potential bias was mitigated by the similarity in the baseline characteristics of the adolescents who reported halitosis and those who did not.

## Conclusion

We found that self-reported halitosis significantly impaired adolescents' OHRQoL. A qualitative study may help to further clarify the role self-reported halitosis plays in OHRQoL of adolescent students in Nigeria.
